# Medical Items

**Published:** 1895-08

**Authors:** 


### *MEDICAL ITEMS.*

---

THE American Academy of Railway Surgeons will meet in Chicago, September 26th.

---

DR. EDWARD R. PALMER, of Louisville, died July 5th. While riding his bicycle Dr. Palmer collided with another rider, fell, and sustained a fracture of the head, from which he died almost instantly.

---

THE Third International Congress of Physiologists will be held at Bern, Switzerland, September 9th to the 13th. Those wishing to attend or contribute papers should address Dr. Frederic S. Lee, Secretary American Physiological Society, Columbia College, New York City.

---

A CORRESPONDENT sends us the following clipping from a Chicago paper: "Dr. Charles J. McIntyre, Chicago, Ill., who recently returned from a visit to Scotland, has been appointed demonstrator of operatic obstetrics in the Chicago College of Physicians and Surgeons," and adds, "There is room for improvement in the music of the average lying-in-chamber, but Dr. McIntyre is now going to make it all right."

---

A MAN at Rochester, New York, accidentally received an electric shock of nearly 3,000 volts, almost double the strength of the current usually employed in the art of electrocution. The D'Arsonval method of resuscitation by artificial inflation of the lungs was immediately begun, and the man revived after an hour. This case apparently lends color to the theory of some that electricity will not kill if proper methods are immediately employed to restore the victim.

